# High mobility group box 1 was associated with thrombosis in patients with atrial fibrillation

**DOI:** 10.1097/MD.0000000000010132

**Published:** 2018-03-30

**Authors:** Qiwen Xu, Lin Bo, Jiaxin Hu, Jin Geng, Yuhan Chen, Xuelin Li, Fu Chen, Jie Song

**Affiliations:** a Department of Cardiology, Nanjing Drum Tower Hospital, Nanjing Medical University; b Department of Cardiology, Nanjing Drum Tower Hospital, Medical School, Nanjing University, Nanjing, Jiangsu, China.

**Keywords:** atrial fibrillation, HMGB1, MyD88, TF, thrombus

## Abstract

**Introduction:**

High mobility group box 1 (HMGB1) is a member of the HMGB family that is involved in inflammatory disease-related thrombosis. We hypothesize that HMGB1 and its downstream factors are associated with thrombosis in atrial fibrillation (AF).

**Materials and methods:**

Our experimental materials were the left atrial appendage (LAA) tissues from patients undergoing valve replacement. The samples were divided into 3 groups: a sinus rhythm group (n = 15), an AF(+)thrombus(−)group (n = 15), and an AF(+) thrombus (+)group (n = 15). The expression of HMGB1, Toll-like receptor 4 (TLR4), advanced glycation end product (RAGE), myeloid differentiation factor 88 (MyD88), nuclear factor κB (NFκB), p-NFκB, and tissue factor (TF) were detected by Western blot and immunohistochemical (IHC) staining. The expressions of interleukin-1 beta, interleukin 6, and tumor necrosis factor-alpha were detected by quantitative real-time PCR.

**Results:**

The Western blots revealed significantly higher expressions of HMGB1, MyD88, p-NFκB/NFκB, and TF in the AF(+)thrombus(+) group than in the other 2 groups. However, no differences in TLR4 or RAGE expression were found between the groups. IHC staining also revealed higher expressions of HMGB1 and TF in the AF(+)thrombus(+) group. The increased mRNA expressions of classic inflammatory factors (i.e., interleukin-1 beta, interleukin 6, and tumor necrosis factor-alpha) in AF(+)thrombus(+) group further validated the correlation between inflammation and thrombi in atrial fibrillation.

**Conclusions:**

HMGB1 was associated with thrombosis in patients with AF via the MyD88/NFκB pathway after adjustment for cardiac and extra cardiac inflammation variables.

## 1 Introduction

Atrial fibrillation (AF) manifests as a highly prevalent sustained arrhythmia in clinical practice and thrombosis is a main complication of AF.^[1]^ Anticoagulation therapy is the principal treatment for atrial fibrillation (AF), but this treatment still has some limitations that result from economic or medical problems. Currently, several studies have identified inflammatory factors as mediators that promote platelet activation, impair endothelial function, and lead to a prothrombotic state.^[2–4]^ Widespread tissue inflammation accompanies the AF pathological process. Whether inflammation is a key factor that contributes to thrombosis in AF requires further elucidation. We look forward to identifying an ideal anti-inflammatory therapeutic target to affect thrombogenesis in AF.

High mobility group box 1 (HMGB1) acts as a member of the high mobility group box family, which is important in inflammatory diseases, such as sepsis, arthritis, atherosclerosis, asthma, and acute pancreatitis in addition to endo-luminal vascular interventions.^[5–9]^ Intracellular HMGB1 mainly regulates the maintenance of chromatin structures of the DNA in normal conditions,^[5]^ and it is released through the cytoplasm to the extracellular region in the presence of appropriate stimuli.^[10–12]^ These appropriate stimuli include apoptosis, necrosis, anoxia, ischemia/reperfusion, and the activation of immune cells through cytokines or Toll-like receptors. Extracellular HMGB1 is involved in the pathogeneses of many diseases via its binding to various membrane receptors, including advanced glycation end products (RAGE) and Toll-like receptors (TLRs).^[13]^ Myeloid differentiation factor 88 (MyD88) is recruited after such binding. This process leads to the translocation of NFκB and the activation of effectors such as tissue factor (TF).^[14,15]^

HMGB1 was confirmed to promote thrombosis. Previous studies have considered HMGB1-associated thrombosis to be primarily associated with platelets, neutrophil extracellular traps (NETs), and endothelial cells. HMGB1 can contribute to platelet adhesion and aggregation to increase pale thrombi. Additionally, HMGB1 contributes to the activation of coagulation factors, fibrin formation, and increases in red thrombi. Several reports have demonstrated that platelet activation and thrombosis development are mediated by the binding of HMGB1 and TLRs or RAGE, which is followed by MyD88 recruitment and NFκB activation in platelets.^[14,15]^ Additionally, NETs can interact with platelets to promote the thrombosis induced by HMGB1. Upon the occurrence of endogenous danger, neutrophils are stimulated to provide their nucleosome material and form the DNA–histone scaffolds that are termed NETs. HMGB1 increases thrombin generation through platelet degranulation and the release of procoagulant microparticles upon the involvement of NETs.^[16]^ It has been reported that HMGB1 can activate MT1-MMP through RAGE, which leads to NFκB phosphorylation and TF protein upregulation in human aortic endothelial cells.^[17]^

HMGB1 is associated with the development of AF. Previous reports have demonstrated that the serum HMGB1 concentration is increased in patients with paroxysmal AF or persistent AF compared with those with normal sinus rhythm (SRs).^[18]^ A HMGB1 gene polymorphism had been found to be associated with the risk of postoperative AF after coronary artery bypass graft (CABG) surgery.^[19]^ HMGB1 is a protein that is associated with inflammation and thrombi in other diseases. An increasing number of studies have proposed that AF patients are susceptible to thrombosis. Structure-related blood rheology, endothelial activation or dysfunction, abnormal platelet activation, decreased fibrinolytic activity, and the production of TF by monocytes may strengthen the inflammation-related prothrombotic effect in AF.^[4,20]^ Inflammatory cytokines and chemokines, such as CRP, IL6, and soluble CD40L, can induce TF expression.^[4]^ Recently, our group reported that TF is increased in the LAAs of AF patients with thrombi.^[21]^ Additionally, Sugimoto et al^[17]^ revealed that HMGB1 can upregulate TF protein expression through the RAGE/NFκB pathway in human aortic endothelial cells. We proposed the reasonable hypothesis that HMGB1 is related to thrombi in AF.

In this study, we focused on demonstrating that HMGB1 may be an ideal therapeutic target. However, whether HMGB1 is involved in thrombogenesis in AF has not been demonstrated. We therefore assessed HMGB1 expression in the atria of patients with and without thrombosis to explore the role of HMGB1 and its downstream effectors in AF-dependent thrombosis.

## 2 Materials and methods

### 2.1 Patients and tissue specimens

This study was approved by the ethics committee of Nanjing Drum Tower Hospital Affiliated to Medical School of Nanjing University (Approval No. 201446) and Huaian First People's Hospital (Approval No. 201431). All patients in this study provided informed consent for the publication of the article.

Forty-five patients who underwent valve replacement surgery for rheumatic heart disease (RHD) at the Nanjing Drum Tower Hospital Affiliated to Medical School of Nanjing University and the Huaian First People's Hospital from January 2014 to May 2015 were included. The exclusion criteria for our experiments were hyperthyreosis, sick sinus syndrome, renal dysfunction, detected rheumatic activity, and other active infectious diseases at the time of recruitment. Active rheumatic activity was evaluated via the modified Jones criteria,^[22]^ macroscopic appearance of the heart tissues at the time of surgery,^[23,24]^ and acute-phase reactants (i.e., ESR > 15 mm/h or CRP > 10 mg/L in the laboratories of experimental hospitals).^[25]^ Fresh or obsolete thrombi were found in the left atria or left atrial appendages during intraoperative explorations. The subdivision of the patients into 3 groups was based on the presence or absence of AF and thrombi as follows: a sinus rhythm group (SR, n = 15), an atrial fibrillation without thrombus group (AF(+)T(−), n = 15), and an atrial fibrillation with thrombus group (AF(+)T(+), n = 15). Every patient underwent a routine transthoracic echocardiographic examination before and after the operation. The patients took all of their normal drugs except warfarin until the morning before the surgery. The portions of the tissues that were used for immunohistochemistry were fixed in 4% paraformaldehyde, and the remaining tissues were stored in liquid nitrogen for long-term preservation at −80 °C for Western blot analyses and qRT PCR.

### 2.2 Western blot analysis

The LAA tissues were thoroughly homogenized on ice in lysis buffer containing 1% protease, 5% phosphatase inhibitor, and 5% phenylmethanesulfonyl fluoride from the Total Protein Isolation Kit (KGP2100, KeyGEN BioTech, Jiangsu, China). Protein quantification was performed with a BCA protein assay (Thermo Scientific). First, 5X protein loading buffer was added to the supernatant. The protein lysates were then mixed and heated to 100 °C for 10 minutes. The concentration of the separation gel was 8% to 15%. Next, the proteins were transferred to polyvinylidene difluoride membranes (Pall Corporation, Ann Arbor, MI). The blot was then blocked with 5% bovine serum albumin in Tris-buffered saline with 1% Tween 20 (TBST) for 1 hour at room temperature. Then, the proteins were incubated with the primary antibodies, which included anti-HMGB1 (ab79823, Abcam), anti-TLR4 (sc-293072, Santa Cruz), anti-RAGE (ab38298, AbSci), anti-MyD88 (ab133739, Abcam), anti-NFΚB-P65 (ab21014, AbSci), anti-NFΚB-P65 (phosphor-ser535; ab11014, AbSci), and anti-TF/F3 (w286, bioWORLD). GAPDH (ab23151, bioWORLD) was used as a loading control. The membranes were incubated with the primary antibodies overnight at 4 °C. Then the membranes were incubated with the HRP-conjugated secondary antibodies for 1 hour at room temperature. Antibody binding was visualized with ECL reagents (Millipore) and the results were quantified using Image-Pro Plus 6.0 software.

### 2.3 Immunohistochemistry staining

The tissues were fixed in formalin, dehydrated in alcohol, embedded in paraffin, and sectioned at 4 μm thickness. The tissues were subsequently incubated with 2% bovine serum albumin in PBS for 30 minutes. The tissues were then incubated overnight at 4 °C with the following antibodies: anti-HMGB1 (ab79823, Abcam), anti-TLR4 (sc-293072, Santa Cruz), anti-RAGE (ab38298, AbSci), and anti-TF/F3 (w286, bioWORLD). The tissues were incubated with the secondary antibodies for 20 minutes, counterstained with hematoxylin and mounted with glycerol gelatin. PBS was used as a negative control.

### 2.4 Quantitative real-time PCR

Total RNA from atrial tissue samples was extracted using TRIzol (Invitrogen) according to manufacturer's protocol. cDNA was generated with a PrimeScript RT reagent kit (Vazyme, China). The mRNA expressions of IL-1β, IL-6, and TNF-α in the samples were analyzed by real-time PCR using a SYBR Premix Ex Taq System (Vazyme, China). The relative levels of the mRNA transcripts for IL-1β (primers: forward TTCGACACATGGGATAACGAGG and reverse TTTTTGCTGTGAGTCCCGGAG), IL-6 (primers: forward CCTGAACCTTCCAAAGATGGC and reverse TTCACCAGGCAAGTCTCCTCA) and TNF-α (primers: forward GAGGCCAAGCCCTGGTATG and reverse CGGGCCGATTGATCTCAGC) were normalized to GAPDH (primers: forward CCTCAAGATCATCAGCAATG and reverse CCATCCACAGTCTTCTGGGT). The relative expressions were determined by the 2^−ΔΔCT^ method using GAPDH as an endogenous control.

### 2.5 Statistical analyses

The data are expressed as the means ± the SDs. One-way analysis of variance was used to examine the differences in the normally distributed and continuous variables between the 3 groups. Kruskal–Wallis tests were used for the non-normally distributed variables. Chi-square analyses were used for categorical variables from the clinical characteristics data. The correlation was analyses were performed with Pearson correlation tests and likelihood ratio tests. Statistical difference was defined by *P* < .05. The results were analyzed with SPSS21.0 software.

## 3 Results

### 3.1 Patient clinical characteristics

The clinical data from our study are presented in Table 1. There were no differences in the demographic data, diabetes, hypertension, or NYHA class between the groups. The patients in the AF groups tended to take more digoxin than those in the SR group (*P* = .003). The AF patients with heart failure may have preferred digoxin to control their symptoms, which could explain the differences in the use of digoxin drugs. Regarding the echocardiographic parameters, the left atrial diameter (LAD) was higher in the AF groups than in SR group (*P* = .002). Additionally, there was a slight tendency towards a decrease in the LAD in the AF(+)T(−) group compared with the AF(+)T(+) group, but no significant difference was found. A typical pathological change of AF is the enlargement of the left atrium which was consistent with our clinical characteristics data. AF patients had larger LAD than SR patients. Other parameters, such as the left ventricular diastolic diameter (LVDd), ejection fraction (EF), and laboratory examination results, were similar among the 3 groups. The CRP and ESR were within the normal ranges, which partially suggest inactive inflammation in these RHD patients, and there were no significant differences between the 3 groups, and the groupings had no effect on the inflammatory markers.

**Table 1:**
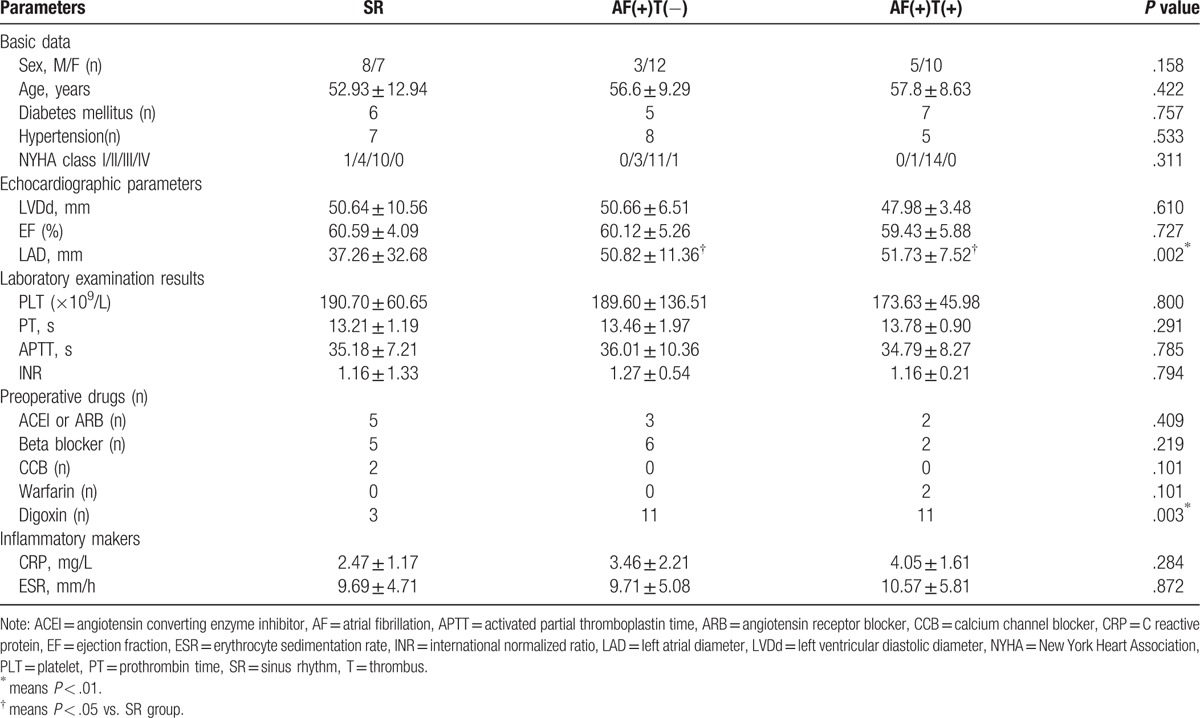
Patient clinical characteristics.

### 3.2 HMGB1 accompanied by TF was associated with thrombosis in AF

**Figure 1:**
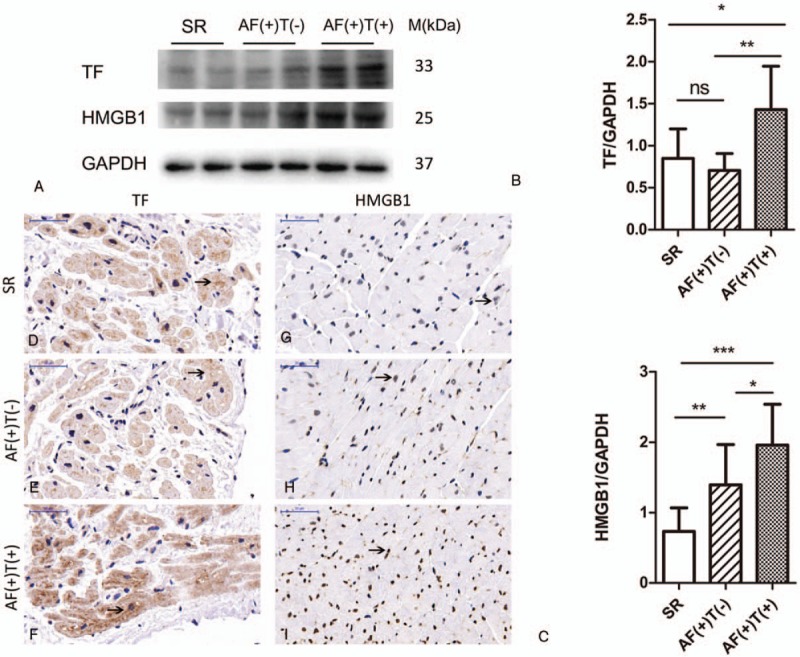
HMGB1 accompanied by TF was associated with thrombosis in AF. (A) Representative Western blot and (B and C) quantitative Western blot analysis of LAAs from patients underwent valve replacement of SR group (n = 15), AF(+)T(−) group (n = 15), AF(+)T(+) group (n = 15) using antibodies against TF, HMGB1, and GAPDH as loading control. Measurements of individual samples were done in duplicate. (D–I) Representative immunohistochemical staining of TF and HMGB1 in SR group, AF(+)T(−) group, AF(+)T(+) group. Arrows indicate TF and HMGB1 protein. Bar = 50 μm. AF = atrial fibrillation, GAPDH = glyceraldehyde-3-phosphate dehydrogenase, HMGB1 = high mobility group box 1, LAAs = left atrial appendages, SR = sinus rhythm, T = thrombus, TF = tissue factor.

TF is also known as coagulation factor III and is an initial component of the coagulation cascade in thrombosis in AF.^[26,27]^ TF has been reported to be expressed in endothelial cells and participates in inflammation associated thrombosis.^[17]^ In previous trials, the expression of TF in AF patients with thrombi has been found to be elevated.^[21,27]^ To analyze whether TF was associated with thrombosis in the LAAs of the AF patients, comparative Western blot analysis with specific antibodies was performed in the present study. The expression of TF was higher in the AF(+)T(+) group than in the AF(+)T(−) group (*P* = .004) and the SR group (*P = *.026). However, the difference between the latter groups was not significant (*P > *.99; Fig. 1A and B).

HMGB1 has been reported to be an upstream regulator of TF in cultured human aortic endothelial cells in a model of atherosclerosis,^[17]^ and it is a key molecule of inflammation and thrombus. We next detected the protein expression of HMGB1 in the present study. In the LAAs, the HMGB1 expression was higher in the AF(+)T(+) group than in the AF(+)T(−) group (*P = *.023) and SR group (*P < *.001). Additionally, the expression of HMGB1 in the AF(+)T(−) group was higher than that in the SR group (*P = *.01; Fig. 1A and C).

To further confirm these results, we used immunohistochemical (IHC) staining to detect the expression of HMGB1 (Fig. 1D–1I). HMGB1 was mainly localized in the nucleus, and it was also expressed in the cytoplasm and cell membranes. TF was mainly localized in the membranes. Consistent with the Western blot analysis, IHC staining revealed that the expressions of HMGB1 and TF were higher in the AF(+)T(+) group than in the other groups. Inflammatory cell aggregation and TF expression were observed around the small vessels (Fig. 1D–1F).

### 3.3 MyD88/NFκB was associated with the thrombosis process

**Figure 2:**
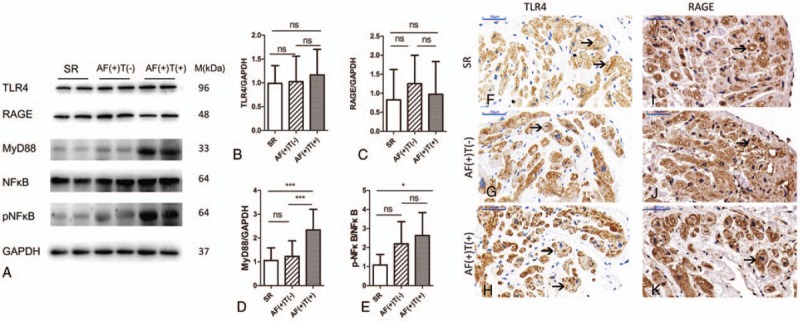
MyD88/NFκB was associated with the thrombosis process. (A) Representative Western blot and (B–E) quantitative Western blot analysis of LAAs from patients underwent valve replacement of SR group (n = 15), AF(+)T(−) group (n = 15), AF(+)T(+) group (n = 15) using antibodies against TLR4, RAGE, MyD88, p-NFκB, NFκB, and GAPDH as loading control. Measurements of individual samples were done in duplicate. (F–K) Representative immunohistochemical staining of HMGB1, TLR4, RAGE, TF in SR group, AF(+)T(−) group, AF(+)T(+) group. Arrows indicate TLR4 and RAGE protein. Bar = 50 μm. AF = atrial fibrillation, GAPDH = glyceraldehyde-3-phosphate dehydrogenase, LAAs = left atrial appendages, RAGE = advanced glycation end product, SR = sinus rhythm, T = thrombus, TF = tissue factor, TLRs = Toll-like receptors,.

The pathway by which HMGB1 affects thrombosis in AF remains unknown. Secreted HMGB1 works by binding to TLRs or RAGE to initiate a MyD88-dependent downstream pathway.^[13]^ We observed the expressions of TLR4 and RAGE in the patients with and without AF and thrombosis (Fig. 2A–C). As presented in Figure 2F–K, TLR4 was localized in the membranes and RAGE was expressed as a secreted protein that was also present in the membrane. Consistent with WB results, there were no significant differences in the expressions of TLR4 or RAGE between the 3 groups.

We further detected the protein expression of MyD88. Similar to the expression of HMGB1, MyD88 was increased in the AF(+)T(+) group compared with the AF(+)T(−) group (*P < *.001) and the SR (*P < *.001) group. There was no significant difference between the AF(+)T(−) group and the SR group (*P = *.763; Fig. 2A and D).

NFκB translocates out of nucleus and is phosphorylated after MyD88 recruitment.^[14,15]^ Next, we detected the phosphorylation of NFκB. As presented in Figure 2E, the p-NFκB/ NFκB ratio in the AF(+)T(+) group was higher than that in the SR group (*P = *.039). However, there were no significant differences among the other pairwise comparisons (*P*_SRandAF(+)T(−)_ = .052 and *P*_AF(+)T(+)and AF(+)T(−)_ > .99).

### 3.4 Linear correlation analyses confirmed the results

**Figure 3:**
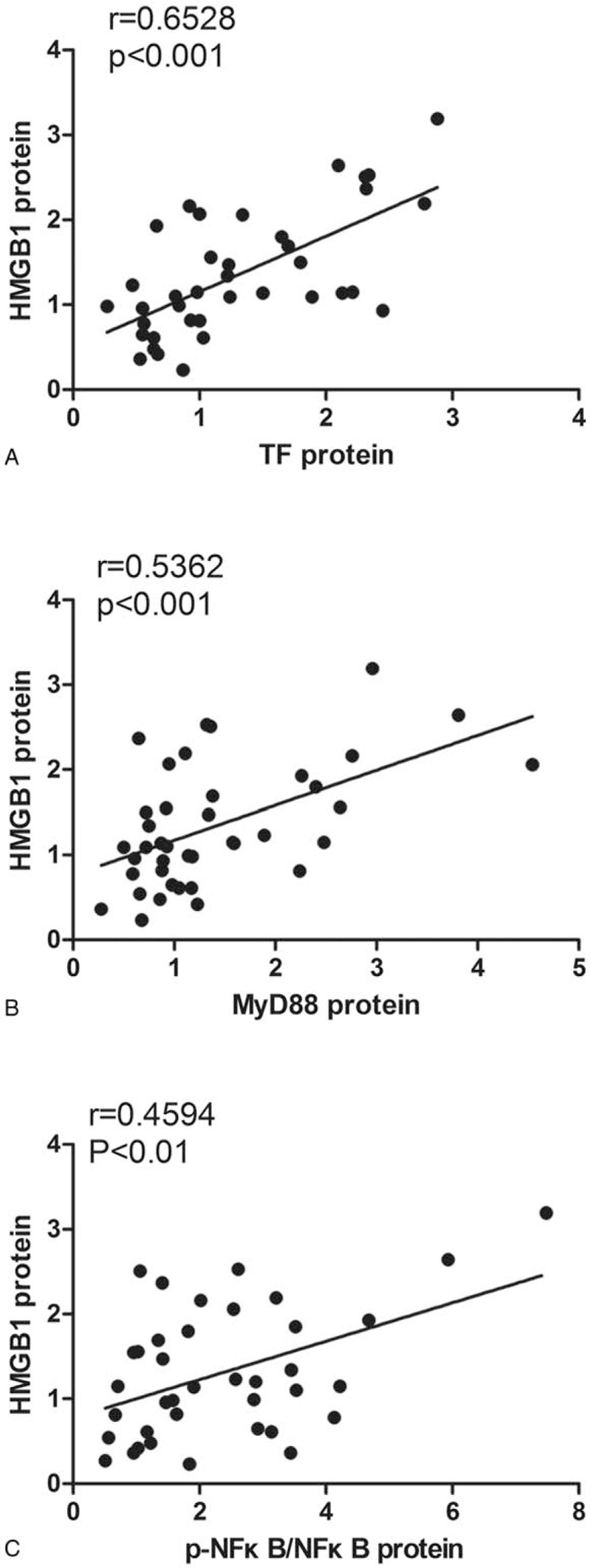
Linear correlation analyses between HMGB1 and other parameters. (A) Relationship between protein expression of TF and HMGB1 (*r* = 0.6528, *P < *.001, n = 45). (B) Relationship between protein expression of MyD88 and HMGB1 (*r* = 0.5362, *P < *.001, n = 45). (C) Relationship between protein expression of p-NFκB/NFκB and HMGB1 (*r* = 0.4594, *P = *.0037, n = 45). AF = atrial fibrillation, HMGB1 = high mobility group box 1, MyD88 = myeloid differentiation factor 88, T = thrombus, TF = tissue factor.

We conducted correlation analyses to ascertain the correlations of HMGB1 with TF, MyD88, and NFκB. There was a significant linear correlation between HMGB1 and TF (*r* = 0.6528, *P < *.001, n = 45, Fig. 3A). As illustrated in Figure 3B, there was a positive correlation between HMGB1 and MyD88 (*r* = 0.5362, *P < *.001, n = 45). Additionally, HMGB1 was also positively and linearly associated with NFκB (*r* = 0.4594, *P = *.0037, n = 45; Fig. 3C).

### 3.5 mRNA expressions of classical inflammatory factors

**Figure 4:**
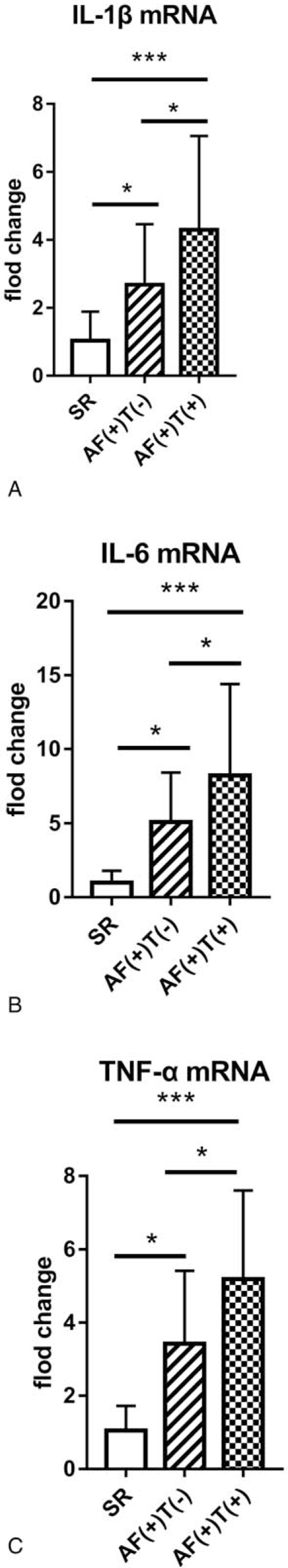
mRNA expressions of classical inflammatory factors normalized to GAPDH. (A) mRNA expression of IL-1β (*P*_SR and AF(+)T(−)_ = .0403, *P*_SR and AF(+)T(+)_ < .001, *P*_AF(+)T(−)and AF(+)T(+)_ = .0367); (B) mRNA expression of IL-6(P_SR and AF(+)T(−)_ = 0.0219, *P*_SR and AF(+)T(+)_ < .001, *P*_AF(+)T(−)and AF(+)T(+)_ = 0.0439); (C) mRNA expression of TNF-α (*P*_SR and AF(+)T(−)_ = .0240, *P*_SR and AF(+)T(+)_ < 0.001, *P*_AF(+)T(−)and AF(+)T(+)_ = .0416). AF = atrial fibrillation, GAPDH = glyceraldehyde-3-phosphate dehydrogenase, IL-1β = interleukin-1 beta, IL-6 = interleukin 6, T = thrombus, TNF-α = tumor necrosis factor-alpha.

To clarify the correlation between inflammation and thrombosis in atrial fibrillation, we detected the mRNA expressions of classic inflammatory molecules (i.e., IL-1β, interleukin-1 beta; IL-6, interleukin 6; TNF-α, tumor necrosis factor-alpha). As illustrated in Figure 4, the expressions of the 3 mRNAs were higher in the AF(+)T(−)group than in the SR group, and the AF(+)T(+) group exhibited the highest levels of expression (IL-1β: *P*_SR and AF(+)T(−)_ = .0403, *P*_SR and AF(+)T(+)_ < .001, and *P*_AF(+)T(−)and AF(+)T(+)_ = .0367; IL-6: *P*_SR and AF(+)T(−)_ = .0219, *P*_SR and AF(+)T(+)_ < .001, and *P*_AF(+)T(−)and AF(+)T(+)_ = .0439; TNF-α: *P*_SR and AF(+)T(−)_ = .0240, *P*_SR and AF(+)T(+)_ < .001; and *P*_AF(+)T(−)and AF(+)T(+)_ = .0416).

## 4 Discussion and conclusions

In this study, we found that HMGB1 expression was significantly increased in AF patients with thrombi. Additionally, we observed elevated expressions of its downstream signaling molecules MyD88 and p-NFκB/NFκB, which indicated that HMGB1 may play a crucial role in thrombogenesis via the MyD88/NFκB pathway in AF patients. Moreover, the mRNA expressions of classic inflammatory factors in the 3 groups further confirmed the results.

We enrolled 45 patients with rheumatic heart disease who underwent valve replacement in our study. Western blots and immunohistochemistry were employed to explore the associations of HMGB1 and downstream pathway with thrombi in AF. The expressions of classic inflammatory factors in 3 groups further validated the correlation between inflammation and thrombi in AF. As illustrated in Virchow's triad, abnormal changes in endothelial function, blood flow and blood constituents contribute to the propensity for thrombogenesis in AF.^[28]^ TF was a key component of the coagulation cascade activation that influenced the blood constituents. Several reports have demonstrated the positive link between TF and AF.^[26,27]^ TF plays a key role in thrombosis in AF and acts downstream of HMGB1 in other diseases.^[17]^ In the present study, the TF expression in the LAA tissues was increased in the AF patients with thrombi, while no significant difference in the SR or AF(+)T(−) group was observed. We found that the expression of HMGB1 accompanied by TF was increased in the AF patients with thrombi. Moreover, the expressions of MyD88 and p-NFκB/NFκB were consistent with that of HMGB1. Linear association analyses indicated positive and linear associations of HMGB1 with MyD88 and NFκB. These data suggested that HMGB1 promotes thrombosis via MyD88/NFκB. However, these analyses revealed no significant differences in the expressions of TLR4 or RAGE between the 3 groups. These findings may have resulted from their synergistic action or the involvement of other TLRs. Several studies have investigated the role of TLR4 in endothelial function. Yang et al^[29]^ reported that low shear stress upregulates TLR4 expression in the endothelial cells. Blood stasis and low shear rate are the classic characteristics of AF. Katoh et al^[30]^ also reported that TLR4 plays roles in atrial endothelial impairment and atrial thrombogenesis in a mouse TAC model, and an elevated expression of TLR4 has been observed in the right atrial tissues of AF patients with congestive heart failure. No published data have revealed the relationship between RAGE and thrombosis in AF. The absence of suitable models for AF is a hindrance to further studies.

Not only HMGB1 gene polymorphism was associated with AF risk,^[19]^ but AF gene polymorphism and HMGB1 were also correlated. Chromosome 7q31 (rs3807989) in CAV1 was an atrial fibrillation susceptibility locus which was identified in a genome-wide association study (GWAS) in individuals of European ancestry.^[31–33]^ CAV1 selectively expressed in the atria and encodes a cellular membrane protein caveolin-1 which was involved in signal transduction.^[34]^ Knockout of CAV1 was associated with dilated cardiomyopathy.^[35]^ It was demonstrated that TLR4 and CAV1 were downstream regulators of HMGB1 in hypoxic trophoblasts in the study of preeclampsia.^[36]^ Phosphorylation of CAV1-Y14 was associated with increased HMBG1 in ventilator induced lung injury.^[37]^ HMGB1 induces the transcytosis of albumin via RAGE-dependent CAV-1 phosphorylation in endothelial hyperpermeability.^[38]^ More thorough research on genetics will be necessary.

IL-6, IL-1β, and TNF-α are proinflammatory cytokines that act in various immune and inflammatory processes. Serum IL-6 is associated with AF recurrence after electrical cardioversion or catheter ablation. In the general population, there is a positive correlation between IL-6 and the risk of atrial fibrillation.^[39]^ Using novel custom-made proteomics, the potential role of IL-6 in the development of AF has further been confirmed.^[40]^ Our previous experiment demonstrated that the protein expression of IL-1 in the atrial tissues of AF patients is higher than that in normal sinus rhythm subjects and even higher in AF patients with thrombi.^[41]^ Sun et al^[42]^ elucidated the mechanism of action of IL-1 in AF; inflammatory macrophages are polarized to increase the electrical remodeling of the atrium via the secretion of IL-1β in animal models. Ren et al^[43]^ revealed that TNF-α affects AF by participating in atrial structure, electrophysiological activity, contraction and remodeling.

When RHD patients exhibit rheumatoid activity or a systemic inflammatory response, inflammatory mediators will be excessively activated, and the coagulation system will be excessively activated. In this study, the patients with rheumatic activity and systemic inflammatory responses were excluded to avoid influencing the experimental results.

Due to the difficulty of modeling, all of these conclusions are based on observational results. The main limitation of our study was the relatively small sample size, which may have led to false-negative statistical results. We cannot explain why there were no significant differences in TLR4 or RAGE at present, and the roles of these receptors may require further investigation. Because the experimental materials were the left atrial appendages of patients who underwent valve replacement, there were no further reduction or over-expression experiments to confirm the results. Furthermore, these patients may not represent nonvalvular atrial fibrillation patients. If possible, we will perform a follow-up experiment with more patients by developing large trial and test groups.

Our study found a correlation between atrial thrombosis and inflammation in patients with AF. Traditionally, anticoagulation acts as an important strategy for preventing embolisms and strokes in patients with atrial fibrillation. Based on our experiments, it can be further assumed that controlling inflammation is a strategy for anticoagulation.

In conclusion, HMGB1 was related to thrombosis in patients with AF via MyD88 recruitment and NFΚB phosphorylation after adjusting for cardiac variables and extra-cardiac inflammation.

## Author contributions

**Conceptualization:** J. Song.

**Data curation:** J. Geng, Q. Xu.

**Formal analysis:** Q. Xu.

**Funding acquisition:** J. Song.

**Investigation:** F. Chen, L. Bo.

**Methodology:** F. Chen, J. Hu, L. Bo.

**Project administration:** F. Chen, J. Hu, J. Song.

**Resources:** J. Hu, J. Geng, X. Li, Y. Chen.

**Software:** J. Geng, Y. Chen.

**Supervision:** J. Geng, Y. Chen.

**Validation:** X. Li.

**Visualization:** J. Song, X. Li.

**Writing – original draft:** Q. Xu.

**Writing – review & editing:** Q. Xu.
